# A narrative review on the use of probiotics in several diseases. Evidence and perspectives

**DOI:** 10.3389/fnut.2023.1209238

**Published:** 2023-07-10

**Authors:** Daniela Campaniello, Antonio Bevilacqua, Barbara Speranza, Angela Racioppo, Milena Sinigaglia, Maria Rosaria Corbo

**Affiliations:** Department of Agriculture, Food, Natural Resources and Engineering, University of Foggia, Foggia, Italy

**Keywords:** probiotics, disease, clinical trials, effects, genera

## Abstract

Gut microbiota is a complex ecosystem, strictly linked to health and disease, as a balanced composition (referred as eubiosis) is necessary for several physiological functions, while an unbalanced composition (dysbiosis) is often associated to pathological conditions and/or diseases. An altered microbiota could be positively affected and partially restored through probiotic supplementation, among others. This review addresses the effects of probiotics in several conditions, used as case-studies (colorectal cancer, neuro-psychiatric diseases, intestinal diseases, obesity, diabetes, metabolic syndrome, immune system, and musculoskeletal system disorders) by pointing out the clinical outcomes, the mode of action, mainly related to the production of short chain fatty acids (SCFA), the impact of probiotic dose and mode of supplementation, as well as trying to highlight a hit of the most used genera.

## 1. Introduction

Since 2001, Lederbergh and McCray highlighted the importance of microorganisms inhabiting the human body in health and disease; in fact, a close connection between the “state of health” of microbial communities and human health was recognized as a milestone (1, 2). Nowadays, “the assemblage of microorganisms (bacteria, archaea, eukaryotes, and viruses) present in a defined environment” is called Microbiota (3) and its composition changes according to the surrounding environment. In particular, the microbiota of the gastro-intestinal tract, generally known as gut microbiota, is a complex ecosystem composed of fungi, viruses, and bacteria, adapted to live on the mucus surface of the intestine or in its lumen, affected, among others, by the modality of childbirth (vaginal vs. cesarean), initial nutrition (breastfeeding vs. formula) and by the guest genotype (4).

The microbial ecosystem balance is called eubiosis and this status allows to perform several functions (nutritional, immunological, preventive actions, etc.); but, if this balance is lacking or altered, there is a condition of “dysbiosis.” Dysbiosis status is often associated to various diseases, such as asthma, chronic intestinal diseases, obesity, diabetes mellitus, psychiatric disorders, and many others (5). Several factors, such as antibiotics, smoking, alcohol, a sedentary life, diets low in fiber, poor chewing, psychophysical stress, chemotherapy, or abuse of drugs (laxatives, antidepressants, sleeping pills, analgesics) heavily affect microbiota balance and could lead to a dysbiotic status (6). An altered microbiota could be positively affected and partially restored through correct diet, and physical activity, although sometimes a supplementation of probiotics and/or prebiotics (e.g., fibers) could be necessary (7).

**Figure 1:**
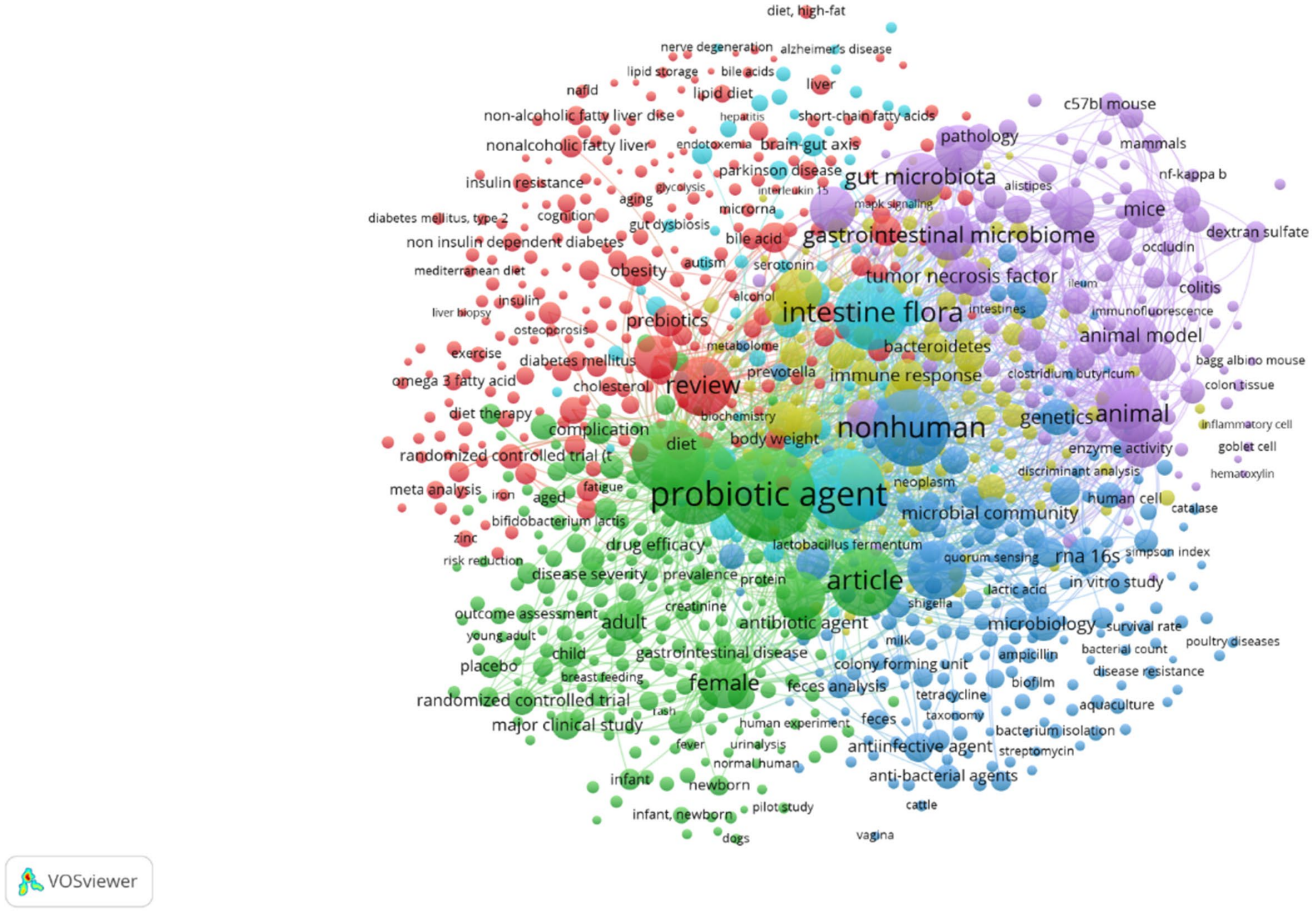
Clustering and most frequent keywords for the research papers and reviews published in 2022 and 2023 on the effects of probiotics on several disease. Elaboration through the software VosViewer.

**Figure 2:**
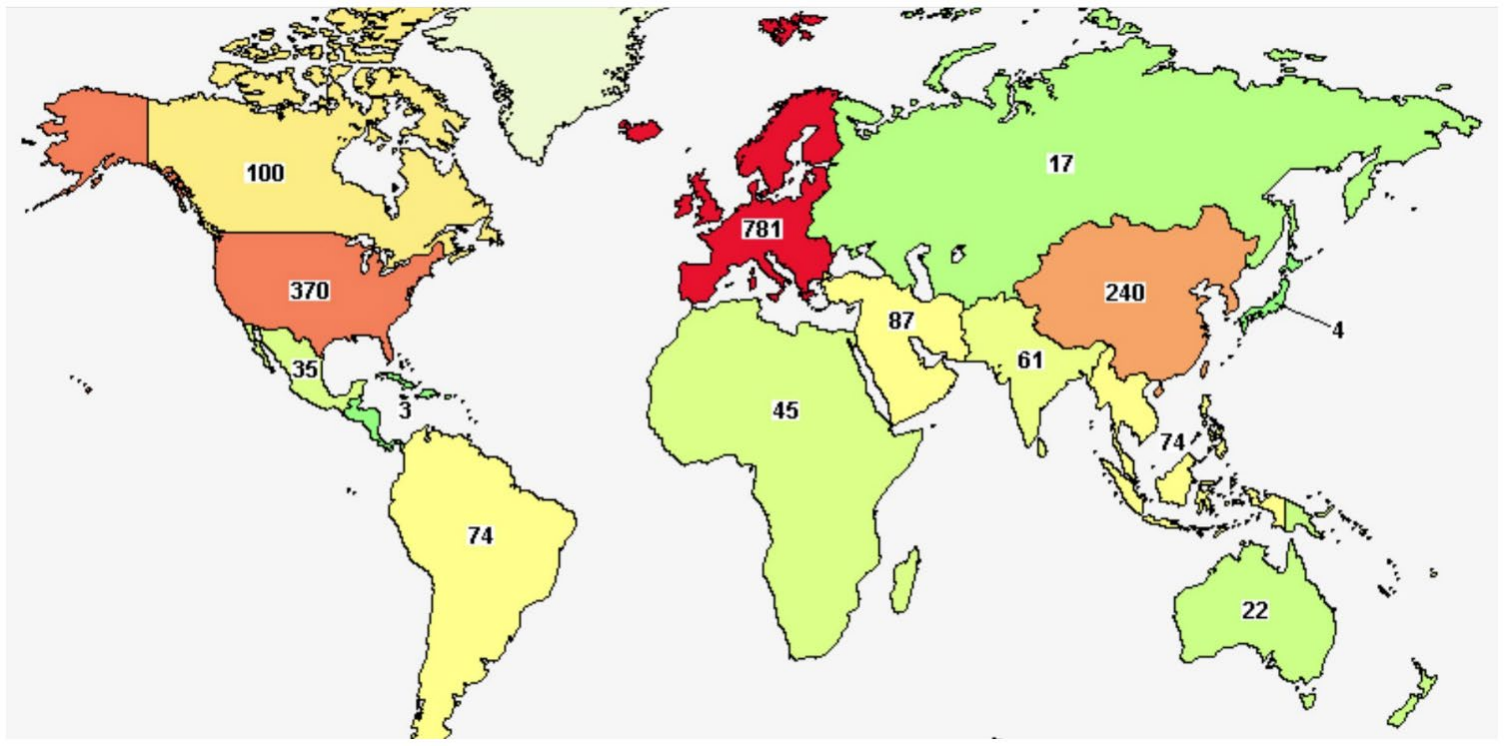
Studies on probiotics on clinicaltrials.gov.

According to the definition of Food and Agriculture Organization/World Health Organization (8), slightly modified by Hill et al. (9), probiotics are “Live microorganisms which when administered in adequate amounts confer a health benefit on the host.” They represent a strategy to treat intestinal dysbiosis, as they could exert some important functions, that is (i) anti-inflammatory activity, essential for maintaining the immune response; (ii) to prevent the colonization by pathogenic microorganisms thanks to the physical barrier function; (iii) to produce antimicrobial substances (10). Thousands of authors studied probiotics and their effects on a wide variety of conditions; a search done on Scopus using two keywords (probiotics and disease) revealed for 2022–2023 more than 4,000 papers (research papers or reviews). The analysis of keywords and abstracts through VosViewer, a tool for networking and clustering of citations and reference details, pointed out a cluster linked to the effects of probiotics on many diseases (red clusters in Figure 1), including among others diabetes, liver diseases, cancer, neurological diseases, obesity etc., thus suggesting the interest toward this topic, also stressed by an overview on clinicaltrials.gov. When the search on this database was done (April 2023), there were more than 2000 items, addressing more than 900 conditions, mainly in Europe and United States (Figure 2).

The papers available on PubMed, and Scopus have some common keywords (intestinal flora, gut microbiota, microbiome) and generally postulate that the beneficial effect of probiotics relies upon the modulation of gut microbiota. In addition, another mode of action of probiotic into the gut is connected to the improvement of gut barrier mucosa; in fact, both an eubiotic gut microbiota and probiotics act at the level of signaling pathways, thus they cause an increase of the mucus, an enhanced production of defensins and proteins in the tight junctions (11). Finally, probiotics, could act on the immune systems, through its direct modulation or indirectly acting on gut microbiota.

It has been reported that 70% of immune cells are in the intestine, mainly in the small bowel, where they constitute the gut associated lymphoid tissue (GALT) (11), thus suggesting that gut is the main site of interaction between host immune systems and commensal microorganisms, either positive or pathogenic. Generally, the activation of the immune system is first based on the recognition of PRRs (pattern recognition receptors) by the microbial associated molecular patterns (MAMPs); MAMPs are components of microbial surface able to interact with the gut epithelium and stimulate the cells of the gut immune system at the lamina propria level (11). Therefore, T lymphocytes are activated, and helper T lymphocytes (Th) are differentiated, by favoring pro- or anti-inflammatory cytokines production (11).

Generally, an eubiotic gut microbiota and probiotics positively affect both host’s innate and adaptive immunity (12); concerning innate immunity, gut microbiota acts both locally and systemically, by influencing the development and function of antigen presenting cells (APCs), neutrophils and other innate cell types (12). Moreover, it has been reported the ability of gut microbiota and of some probiotics to affect innate immunity outside the gut milieu, for example by promoting the attenuation of inflammation processes at local levels (13, 14). There is also a role on adaptive immunity, due to the effect in the development of the most important subtypes of CD4^+^ T cells (or helper T cells, which are lymphocytes coordinating the response to diseases), that is Th1, Th2, Th17 and T_reg_ (12, 15). In addition to T cells, an eubiotic gut microbiota could influence B cell maturation and immunoglobulin production (16).

The mechanisms by which gut microbiota and probiotics influence immune system include the production of various compounds; SCFA (short chain fatty acids; butyrate, acetate, formate), indole derivatives, and bile salts are, among others, the most important. An extensive description of the effects of indole derivatives on gut microbiota is in the review of Ye et al. (17); however, it is worth mentioning that indole derivatives, produced by gut microbes and some probiotic strains (e.g., *Limosilactobacillus reuteri*) through the metabolism of tryptophan are crucial, because they enhance intestinal epithelial cell function by regulating several genes involved in mechanical barrier formation. Moreover, they increase mucin and goblet cell secretion products, responsible of barrier of gut mucosa, and reduce the impact of possible pathogens (17).

SCFA are produced through the fermentation of non-digestible carbohydrates and amino acids in the colon and play a major role in maintaining the barrier function of gut (18). They are absorbed by the colonocytes and used as fuel for the colonic mucosal epithelial cells (19), but at the same time they directly act on gut mucosa; for example, butyrate contributes to reduce oxidative stress, thus stabilizing gut mucosa and reducing the translocation of LPS (Lipopolysaccharide) (12). Also, bile salts are essential for immunity in a bidirectional crosstalk between host and microbiota. Primary bile salts, or host-derived bile salts, shape and modify the composition of microbiota, generally reducing the levels of Gram-negative bacteria; while those synthesized by microbiota contribute to a further modulation of microbiota itself and act on both innate and adaptive immunity, for example by reducing the levels of pro-inflammatory cytokines, or enhancing T_reg_ cells differentiation (20).

SCFA and derivatives from tryptophan could also play a significant role in reducing inflammatory status. SCFA bind to specific receptors on intestinal epithelial cells, thus they inhibit NF-κB pathway, T_reg_ cell suppression, and pro-inflammatory cytokine production by neutrophils and macrophages (21). For example, butyrate could control gut inflammation through the induction of T_reg_ cell differentiation (22). In addition, tryptophan (deriving from diet) and indolic acid derivatives (for example IPA, indole-3-propionic acid) bind to receptors expressed on immune cells, promote IL-10 production with anti-inflammatory activity and decrease TNF-α release (21).

It is worth mentioning that the ability of potential probiotics to modulate the immune system and ameliorate inflammatory status depend on the strains and a comprehensive overview of the effects at species level is missing (23). Other topics missing in the literature are the technological aspects of the problems (production and dose of probiotics). Therefore, the main goal of this paper is an overview of the effects of probiotics on some representative conditions, addressing some key-points, like the clinical effects, and the mode of action of probiotics, if available; the elucidation of aspects common to all strains of a species, and finally a focus on the importance of a correct dose.

There are many pathological conditions; however, by authors’ choice only research papers and some representative conditions were chosen, as best models for future studies, that is colorectal cancer, neuro-psychiatric diseases, intestinal diseases, obesity, diabetes, metabolic syndrome, which are probably the most addressed topics in the literature, along with two minor issues (immune system, and musculoskeletal system disorders), which are promising ways but with a few evidence.

For each pathological conditions, the effects of probiotics are described, and the list of studies and outcomes is in reported, along with the kind of probiotic, or the probiotics mix, the target of the study (humans or animal model), and the achievable and measurable outcomes (Supplementary Table S1).

## 2. Colorectal cancer

Colorectal cancer (CRC) is the most frequent neoplastic form of the gastrointestinal tract; its incidence is experiencing a progressive increase, due to a gradual aging of the population, the adoption of sedentary lifestyle, and unbalanced diets (24), as also suggested by the higher incidence rates in Australia and New Zealand, North America, and Europe (25). Although it is a multi-etiological condition, it should be considered the genetic susceptibility of each individual, as well as some environmental factors connected to carcinogenesis, like caloric intake, obesity, alcohol or smoking (26–37). Focusing on gut microbiota, CRC patients often develop a dysbiosis due to the use of antibiotics, radiation therapy, and chemotherapy, and their gut microbiota is characterized by an increased pathogenic bacteria abundance, decreased SCFA-producing bacteria and SCFA levels (38, 39) and butyrate seems the most affected compound, as it could be successfully used as a potential biomarker of CRC risk or as an early warning signal of the disease onset (40). Conversely, high levels of SCFA have antineoplastic properties, due to a combination of several mechanisms, like the downregulation of the canonical Wnt signaling pathway linked to colonic carcinogenesis, the limitation of proliferation and migration of neoplastic cells, the suppression of tumor angiogenesis, the induction of apoptosis and the promotion of neoplastic colonocytes differentiation (40).

**Figure 3:**
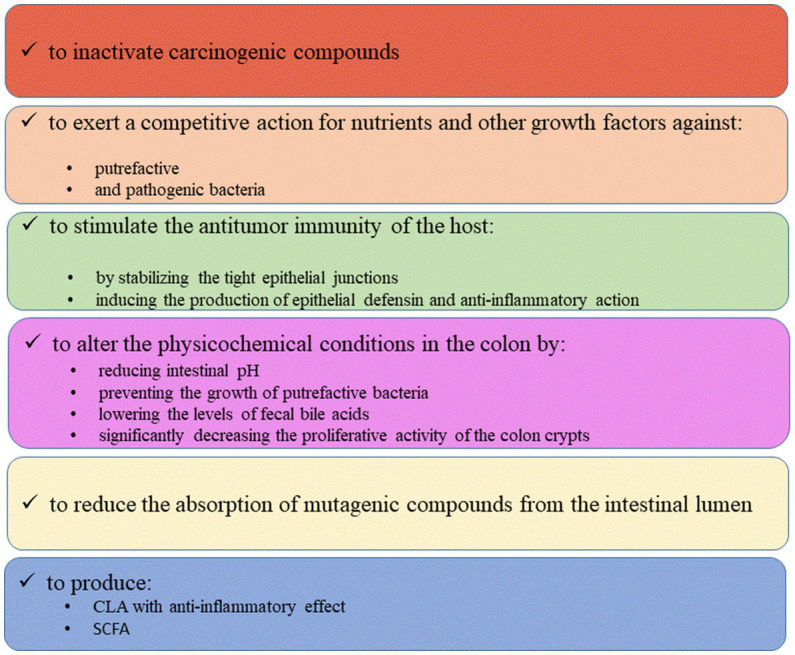
Probiotic effects on CRC.

Although the production of SCFA probably exerts a major role in the anti-carcinogenic activity of probiotics, there are also some other direct and indirect effects, briefly summarized in Figure 3, including the ability to catch and adsorb carcinogenic compounds, as well as by stimulating host’s antitumor activity through the stabilization of the tight junctions or the production of defensins. Other effects include the antagonistic activity toward putrefactive microbiota and the creation of a microenvironment into the colon unfavorable for the carcinogenesis.

Many research papers and clinical trials have addressed the role of probiotics in the CRC onset and/or mitigation and a comprehensive overview of the most important trials is in the paper of Hou et al. (40); Supplementary Table S1 shows some relevant studies. In particular, *Bacteroides fragilis* exerts anti-inflammatory and anticancer effect, as it can alter the composition of the microbiota, inactivating carcinogenic compounds, competing with pathogens or CRC-promoting bacteria and stimulating the immune response (41); similar effects could be observed for *Lactobacillus acidophilus* MTCC 5401 (42) and *Faecalibacterium prausnitzii* (43), while *Lactococcus lactis* subsp. *cremoris C60* and *Lacticaseibacillus casei* ATCC334 probably exerted a preventive and an inhibitory effect on the cells responsible for CRC (44, 45). In addition, an emergent butyrate-producing probiotic, *Butyricicoccus pullicaecorum* exerted antitumor effect and showed good acid and bile tolerance; it was also able to reduce pathogen population and to prevent necrotic enteritis (41).

Shang et al. (46) demonstrated the effectiveness of a probiotic mix composed of *Bifidobacterium longum*, *Bifidobacterium bifidum*, *L. acidophilus* and *Lactiplantibacillus plantarum* in mice, able to reduce the tendency of CRC cells to migrate in different body tissues. Furthermore, the tumor size in mice feed with probiotic mixture was significantly smaller than the control group. In another study, Dong et al. (47) investigated *Ligilactobacillus salivarius* effect on CRC cells, via oral administration in male mice. The authors reported that probiotic induced the suppression of dimethylhydrazine (DMH) production, both in the early and post-early stages of carcinogenesis. DMH is a potent carcinogen used to induce colon cancers in animals, particularly mice. These results therefore suggest that daily oral administration of *L. salivarius* could effectively prevent CRC carcinogenesis by inhibiting cell proliferation and inducing apoptosis in DMH-induced tumor models.

Probiotics could also counteract dysbiosis occurring in most patients after CRC resection and improve the biodiversity of bacterial biota. In this context, Park et al. (48) observed improvements in postoperative intestinal dysbiosis with the use of probiotics in CRC surgical resection patients. Sixty patients, aged between 18 and 75, with sigmoid colon adenocarcinoma and anterior resection of the same, were divided into two groups: 29 and 31 patients feed with a probiotic mixture (*Bifidobacterium animalis* subsp. *lactis* HY8002, *L. casei* HY2782 and *L. plantarum* HY7712) and the placebo respectively, for 4 weeks. Probiotics led to an increased production of SCFA by colon bacteria, decreased microbes associated with the development of CRC (mainly *Alloprevotella* and *Porphyromonas*) and improved postoperative recovery of patients. Particularly interesting were the data obtained from the measurement of faecal zonulin, a protein that acts on the tight junctions of the intestine, regulating its permeability; high levels are associated with a deterioration of the intestinal mucosa, which does not adequately perform its protective function. The authors found that zonulin significantly decreased in the group fed with probiotic mixture compared to the placebo group.

The efficiency of *L. plantarum* was also observed by Yoon et al. (49); the authors evaluated the effect of *L. plantarum* CJLP243 (isolated from kimchi, a traditional fermented product of Korea) on intestinal function and quality of life toward 36 patients aged 20–75, who have undergone rectal resection and were admitted undergoing the reversal of the ileostomy. Unfortunately, a significant number of patients reported symptoms including diarrhea, fecal incontinence, and other complications. The patients were divided into two groups: 19 and 17 patients who took placebo and probiotic respectively, once a day for the duration of 3 weeks. The results showed that there were no significant differences between the two groups regarding the improvement of symptoms; however, by comparing the post-operative results between the first and third weeks, the administration of the probiotic showed a tendency to improve intestinal function and quality of life.

## 3. Neuro-psychiatric diseases

Many human and animal studies support the idea that gut microbiota plays an important role for cognitive functions, in the regulation of mood and emotions, and in the interpersonal interactions and communications (50). Gut microbiota can modulate brain activity and behavior; therefore, its manipulation can be applied in the treatment of neuropsychiatric disorders such as autism spectrum disorders, depression, etc. (51, 52). The idea that probiotic could positively affect the clinical outcomes of depression was first postulated in 1910 when Hubert J. Norman and Georges Porter Philipps found an improvement in the symptoms after taking lactobacilli (53). Later then, this idea has been confirmed by several studies and clinical trials, although the mode of action of probiotic on behavior and neuro-psychiatric diseases is still unclear, as in some cases symptoms improvement and amelioration are not related to a modification in gut microbiota (53).

Supplementary Table S1 reports 33 scientific articles concerning the effect of probiotics in subjects with neuro-psychiatric diseases. Twelve articles refer to autism (ASD), a neurobiological developmental disorder, characterized by severe and generalized impairment of both communication skills and social interaction. Subjects affected by ASD, especially in children aged 2 to 11 years, show a stereotypical use of movements, language or objects, excessive adherence to routine situations, routines, rituals, and fixation for particular or restricted interests abnormally in duration or intensity (54). The benefits of probiotics depend on the microorganisms. For example, an anti-inflammatory effect was found following the administration of *Bifidobacterium* spp. (55), while improvement of gastrointestinal disorders and neuro-behavioral symptoms was achieved by microbial mixtures composed of several strains of *Bifidobacterium*, *Lactobacillus*, *Streptococcus* genera as well as *L. plantarum* PS128, *Limosilactobacillus reuteri* and *Lacticaseibacillus rhamnosus* GG (56–62). In particular, the effectiveness of *L. plantarum* PS128 relied upon the age of the children, as the best results were obtained on infants (60).

For anxiety and depression, the outputs showed an improvement in the gut microbiota with a reduction in depressive and anxious behavior (63–65). In particular, Abildgaard et al. (64) proposed a mixture of probiotics (*B. bifidum* W23, *Bifidobacterium lactis* W52, *L. acidophilus* W37, *Levilactobacillus brevis* W63, *L. casei* W56, *L. salivarius* W24, *Lactococcus lactis* W19, *L. lactis* W58) as potential treatment strategy in major depressive disorders (MDD) to reduce depressive behavior. Some studies reported improvement in behavioral abnormalities and reduction in the main symptoms of depression in humans, after the administration of strains belonging to the genera *Lactobacillus* and *Bifidobacterium* (66–71). Another possible use of probiotics refers to dementia and cognitive deterioration. The intake of *Enterococcus faecium* together with inulin (72) and *Bifidobacterium breve* A1 (73) improved learning and memory skills, language, attention and orientation in the elderly people. In addition, some studies on animals showed an improvement in the intestinal barrier and spatial learning through the administration of *L. casei* LC122, of *B. longum* BL986 and of *Clostridium butyricum* (74, 75).

For Parkinson’s disease (PD) Tamtajii et al. (76) and Magistrelli et al. (77) observed that *L. acidophilus*, *B. bifidum*, *L. reuteri*, *Limosilactobacillus fermentum* and *L. salivarius* allowed an improvement in MDS-UPDRS (Movement Disorder Society-Unified Parkinson’s Disease Rating Scale) scores and a significant reduction in pro-inflammatory cytokine levels and reactive oxygen species (ROS), with a possible weight of the stage of the disease and sex. In animal models, Barichella et al. (78) showed that the genera *Lactobacillus* and *Bifidobacterium* could improve intestinal integrity and reduce anxiety, depression and stress.

Anorexia nervosa (AN) consists of an altered perception of one’s own body, in particular weight. In fact, people who are in this condition try to keep their body weight as low as possible through a strong dietary restriction, inducing vomiting and practicing intense physical activity. AN most frequently affects young women, although recently it has also targeted men; it can often be associated with psychological problems such as depression, anxiety, low self-esteem, alcohol abuse, and self-harm (79, 80). *L. plantarum* P8 determined a reduction in anxiety and stress (81) while *B. fragilis* reduced gastro-intestinal pains and caused as a secondary effect an increase serotonin production (82); it is not clear if these effects have a connection or are independent outcomes (Supplementary Table S1). In animals, *Lactobacillus* spp. promoted weight gain (83) and improved the behavioral abnormalities in stressed mice involving the microbiota-brain gut axis (84). Moreover, *Akkermansia muciniphila*, considered a potential candidate for improving metabolic disorders associated with anorexia, obesity, diabetes, liver disease, favored the restoration of a compromised intestinal barrier (85).

Probiotics were also studied in relation to the benefits they bring for other diseases affecting the brain systems. For example, the administration of *L. acidophilus*, *B. bifidum* and *B. longum*, improved the cognitive function of Alzheimer’s patients (humans and in animals) (86, 87), while strains of *L. rhamnosus* GG and *B. animalis* subsp. *lactis* Bb12 led to an improvement of the symptoms related to schizophrenia (such as delirium, hallucinations, language, and disorganized behavior, etc.) (88). Furthermore, in women aged 20–40 affected by multiple sclerosis, a mixture of probiotics (*Lacticaseibacillus paracasei*, *L. plantarum*, *L. acidophilus*, *L. delbrueckii*, *B. longum*, *Bifidobacterium infantis*, *B. breve*, *Streptococcus thermophilus*) improved the symptoms by modulating the anti-inflammatory immune response (89). Referring to multiple sclerosis, Altieri et al. (90) in a recent review described how microbiota change in MS patients and proposed probiotics as useful tools to improve the symptoms of MS patients.

## 4. Intestinal diseases

Generally, probiotics could positively impact on gastrointestinal disorders (GI) (abdominal pain or discomfort, swelling and flatulence) through metabolic effects resulting from enzymatic activity and the crosstalk with the central nervous system, by improving gut function (91). In addition, there are several evidence on positive effects on Inflammatory Bowel Disease (IBD) and Irritable Bowel Syndrome (IBS).

Concerning IBD, Ferreira-Halder et al. (43) and Lopetuso et al. (92) highlighted the anti-inflammatory effect performed by *F. prausnitzii* and *A. muciniphila*. *F. prausnitzii* contributes substantially to the health of the intestine and is considered a biomarker not only for human health but also for diagnosis and subsequent treatment (43). On the other hand, *A. muciniphila* has been shown to be effective in immune and metabolic regulation; it ensures increased function of the intestinal barrier showing a direct and beneficial effect on the host’s response. In addition, its use is considered safe if aimed at human studies (93).

In patients with ulcerative colitis, probiotics act as a barrier against harmful microorganisms. A consortium of 8 probiotic strains (VSL3, composed of *L. casei*, *L. plantarum*, *L. acidophilus*, *L. delbrueckii* subsp. *bulgaricus*, *B. longum* subsp. *longum*, *B. breve* and *B. longum* subsp. *infantis*, *Streptococcus salivarius* subsp. *thermophilus*) was effective in maintaining a state of remission (94), while Azad et al. (95) reported that *Lb. acidophilus* restored the balance of inflammatory cytokines and Th17/T_reg_ cells in mice induced colitis, and showed beneficial effects in the prevention of cancer and intestinal inflammation (95).

In addition, several analyses have shown the effectiveness of the administration of probiotics in premature infants, with a reduction of both the development of enterocolitis and the risk of sepsis in old age. In particular, Dermyshi et al. (96) supported the benefits of *L. acidophilus*-*B. infantis* blend.

IBS causes swelling, vomiting, diarrhea, abdominal pain, frequency of stools, and probiotics could improve these symptoms. Two formulations containing different probiotic strains (F1 = *L. acidophilus*, *L. reuteri*; F2 = *L. plantarum*, *L. rhamnosus*, *B. animalis* subsp. *lactis*) were administered to humans, thus gaining a relief in bloating, abdominal pain, constipation, abdominal cramps, and flatulence (97). Similar effects were observed through the administration of *Bacillus coagulans* MTCC 5856 (98), and *L. plantarum* DSM 9843 (99). Other studies reported the improvement of IBS symptoms due to several lactobacilli (100, 101).

## 5. Obesity

Gut microbiota is involved in the control of body weight, energy homeostasis and inflammation states; therefore, it plays an important role in the pathophysiology of obesity. Firmicutes and Bacteroidetes are the two phyla involved in microbial dysbiosis and in the development of obesity. The ratio between these phyla is very important; in fact, Bervoets et al. (102) studied the gut microbiota of 26 overweight and obese children and 27 skinny children and found that obese children have a higher ratio of Firmicutes to Bacteroidetes.

Supplementary Table S1 focuses on some application of probiotics toward overweight and obese subjects. Kadooka et al. (103) administered fermented milk containing *Lactobacillus gasseri* SBT2055 (200 g/day) to 87 overweight adults for 12 weeks. Reductions in visceral and subcutaneous fat, body weight and BMI (Body Mass Index) compared to the control group, were observed. Furthermore, the consumption of yogurts supplemented with capsules, containing 10^9^ CFU of *Lactobacillus amylovorus* and *L. fermentum* by 28 overweight participants, led to a reduction in total body fat mass (104). Regarding gut microbiota, the researchers observed a significant reduction of *Clostridium* cluster IV (for *L. amylovorus* consumption), together with an increase of *Lactobacillus* in both treatments and concluded that when the gut microbial composition is modulated through probiotic consumption, this can positively alter energy metabolism and body composition (104). An additional study on 70 overweight and obese children revealed that a combination of probiotics, prebiotics and vitamins A, E and C for 8 weeks, significantly reduced BMI, waist circumference, waist/hip ratio, LDL cholesterol and triglycerides (105).

Probiotics can reduce cholesterol levels through bile salt hydrolase (an enzyme that hydrolyzes bile salts into amino acid residues and free bile salts). 200 g/day of yogurt containing *S. thermophilus*, *L. delbrueckii* subsp. *bulgaricus*, *L. acidophilus* LA-5, and *B. animalis* BB12 for 9 weeks to 70 women in the third trimester of pregnancy resulted in significant reductions in total cholesterol, LDL cholesterol, and high-density lipoprotein (HDL) cholesterol, as well as serum triglyceride concentrations (106). Probiotic supplementation also reduced blood lipid concentrations (107).

*A. muciniphila* administered to animals led to a reduction in fat mass and body weight; moreover, it favored the restoration of the intestinal barrier function and, if administered to humans, improved inflammation, insulin resistance and blood sugar level (108).

Many authors reported that the action of probiotic toward obesity is mediated by SCFA, which probably could be involved in body weight regulation, and maintenance, as well as in energy intake and expenditure (109–111). Although there are several hypotheses, the most probable mechanism involves the ability of propionate and butyrate to bind to G-protein-coupled receptors in the colon leading to the production of the gut hormones peptide YY and glucagon-like peptide 1, thus influencing satiety and glucose homeostasis (109). In addition, SCFA activate intestinal gluconeogenesis, and the released glucose mediates a signal to brain through portal nerves for satiety and insulin sensitivity, or they can also affect peripheral metabolism in the liver (enhanced lipid oxidation, lower lipid storage), skeletal muscles (increase of glycogen synthesis and reduction of glycolysis), pancreas (increase of insulin and reduction of glucagon synthesis and release) or adipose tissue (reduction of insulin mediated adiposity) (109, 110). The evidence available in the literature suggest that that increasing SCFA production could be a preventive measure to counteract gastro-intestinal dysfunction, obesity, and type 2 diabetes mellitus (109, 110), although longer term trials and data are required, also to elucidate the exact role of the initial imprinting of gut microbiota and how it can respond to probiotic intervention.

## 6. Diabetes

Generally systemic inflammation involve microbiota as it modulates inflammation, interacts with nutrients, influences intestinal permeability, glucose and lipid metabolism, insulin sensitivity and the body’s energy balance. The microbiota of diabetic patients is poorly populated by useful microorganisms (*Bifidobacterium*, *Bacteroides*, *Faecalibacterium*, *Akkermansia*, and *Roseburia*) which have anti-inflammatory activity, are butyrate-producing and are promoters of low intestinal permeability and may have inhibitory activity against carbohydrates-degrading enzymes, reducing postprandial hyperglycemia. On the contrary, there are many microorganisms favoring the production of inflammatory molecules and the alteration of intestinal permeability such as *Ruminococcus*, *Fusobacterium*, and *Blautia* (112, 113). In any case, considering that diabetes is closely linked to food choices and habits, it is certainly essential to make adequate decisions in this regard; for example, an active lifestyle could improve insulin resistance, while taking foods rich in fibers, largely represented by prebiotics, is certainly a positive choice for wise prevention.

Positive effects such as increased insulin sensitivity and improvement of microbial diversity were found following administration of *L. reuteri* DSM 17938 to patients with type 2 diabetes (114).

Toejing et al. (115) administered *L. paracasei* HII01 (50 × 10^9^ CFU/day) to 50 T2DM (type 2 diabetes mellitus) patients to evaluate the effect on glycemia and observed that after 12 weeks fasting blood glucose (FBG) level significantly decreased. Furthermore, probiotics reduced the plasma levels of lipopolysaccharide (LPS), inflammatory markers (TNF-α, IL-6) and C-reactive protein (hsCRP). A reduction in pathogenic microorganisms together with improvement in beneficial bacteria were also observed; therefore, the authors concluded that *L. paracasei* HII01 could play a potential role as an adjuvant treatment in type 2 diabetes.

A potential antidiabetic effect was also observed by using another *Lactobacillus* strain: Wu et al. (116) investigated the performances of *L. rhamnosus* LRa05 on glucose metabolism and gut microbiota in T2DM mice. The treatment with 10^9^ CFU/day of *L. rhamnosus* resulted in a reduction in the fasting blood glucose (FBG) levels (by 53.5%), lowered insulin resistance, alleviated metabolic lipopolysaccharide-related inflammation and relieved hepatic oxidative stress. Further positive effects were found on the gut microbiota composition; in fact, SCFA producing microorganisms, such as *Alloprevotella* and *Bacteroides*, increased with a reduction of proinflammatory microorganisms such as *Odoribacter* and *Mucispirillum* (116).

Manaer et al. (117) reported the benefits of *Lactobacillus* and yeasts on T2DM mice. Probiotics (*Lactobacillus kefiranofaciens*, *L. plantarum*, *Lactobacillus helveticus*, *L. lactis*, *Issatchenkia orientalis*), isolated from traditional fermented cheese whey (TFCW), were used to prepare a mix from camel milk (CPCM) to feed db/db mice. The authors studied how these strains affect gut microbiota, glucose and lipid metabolism, liver and renal functions. CPCM reduced fasting blood glucose (FBG), oral glucose tolerance test and glycosylated hemoglobin HbAlc, increased C-Protein, modulated lipid metabolism and improved liver. Finally, CPCM increased LAB and *Bifidobacterium* population in intestinal tract and decreased *Escherichia*.

Razmpoosh et al. (118) evaluated the effect of 7 probiotics (*L. acidophilus*, *L. casei*, *L. rhamnosus*, *L. bulgaricus*, *B. breve*, *B. longum*, *S. thermophilus*), and 100 mg of fructo-oligosaccharide (FOS) with lactose as carriers, on lipid profile and glycemic control in 60 patients. They were equally divided into 2 groups (group 1 took probiotics and group 2 took a placebo, for 6 weeks). A significant decrease in the fasting plasma glucose (FPG) and increase of high density of lipoprotein cholesterol (HDL-C), was observed. No significant differences in the levels of insulin, triglycerides, total cholesterol, insulin resistance and anthropometric measurements (weight, waist circumference and body mass index).

## 7. Metabolic syndrome

Metabolic syndrome (MetS) is a pathology characterized by an excess in abdominal fat, arterial hypertension, impaired fasting plasma glucose (FPG) or insulin resistance, whose diagnoses and treatments are often similar to those of obesity (119). Supplementary Table S1 lists 6 papers concerning the study of the effect of some probiotics in subjects with MetS.

Corb Aron et al. (108) and Ottman et al. (93) used *A. muciniphila* to evaluate its effect on volunteers with MetS. They observed that the probiotic degrades mucin by stimulating the production of new mucous layer (108) and contributes to immune and metabolic regulation by increasing the intestinal barrier (93). At the same time, the metabolic activity of *A. muciniphila* led to the production of SCFA with beneficial effect to the host and members of the microbiota (93).

Instead *L. plantarum* (120), *L. acidophilus* and some *Bifidobacterium* species (*B. bifidum*, *B. lactis*, and *B. longum*) (121) mainly led to a reduction in blood sugar and cholesterol. In particular, reduction in LDL cholesterol, blood glucose, and homocysteine levels when postmenopausal women were treated with *L. plantarum* for 90 days (120).

## 8. Musculoskeletal system

The role of probiotics in the control of musculoskeletal diseases is a topic of great interest; osteoporosis (characterized by a decrease in bone strength, a low mineral density of the bone tissue, with consequent fragility and aging) (122), osteoarthritis (a non-inflammatory arthropathy involving cartilage and bone remodeling) or bone fragility, and microbiota changes are closely related (123).

It has been demonstrated that the synergistic action of *L. casei* with type II collagen (CII) and glucosamine (GS) (potential prebiotic), administrated to arthritic rats, led to an effective reduction of pain and cartilage destruction. Moreover, a reduced expression of numerous proinflammatory cytokines, resulted (124).

Supplementary Table S1 reports some cases concerning the use of different *Lactobacillus* strains to relieve bone, joint and muscle disorders. The ability of probiotics to reduce pain and cartilage destruction has been highlighted in experiments conducted on animals (125) together with numerous effects, such as antimicrobial, antioxidant, anti-inflammatory (126), the ability to determine an increase in calcium (127) and recovery of joint strength (128) in humans.

Steves et al. (125) and Paul et al. (126) demonstrated that *L. casei* and *L. acidophilus* improved intestinal dysbiosis and the symptoms of rheumatoid arthritis after long-term repeated use thanks to their anti-inflammatory, antimicrobial and antioxidant properties. These microorganisms act symbiotically in the intestine to establish their colonization and consequently increase the integrity of the cell layers of the gastro-intestinal tract, maintain the nutritional support of the host and reduce the severity of inflammatory conditions.

## 9. Immune system disorders

It is known that probiotics can also bring benefits through the modulation of the immune system. Supplementary Table S1 shows 3 articles focused on the effect of probiotics on the modulation of the immune system. Among the most significant results, there are the bactericidal and antitumor effect with production of proinflammatory and anti-inflammatory cytokines in humans, by *E. faecium* (95) and the development of regulatory cells in the gastrointestinal epithelium in animals, by strains of *L. reuteri* (99). Finally, Han et al. (129) treated mice with *L. rhamnosus* HDB1258 and observed that it enhanced the immune response by activating innate immunity. In addition, *L. rhamnosus* suppressed systemic inflammation by increasing the expression ratio of anti-inflammatory cytokines and modulated the microbiota composition.

## 10. Probiotic species, dose, delivery, and production

**Figure 4:**
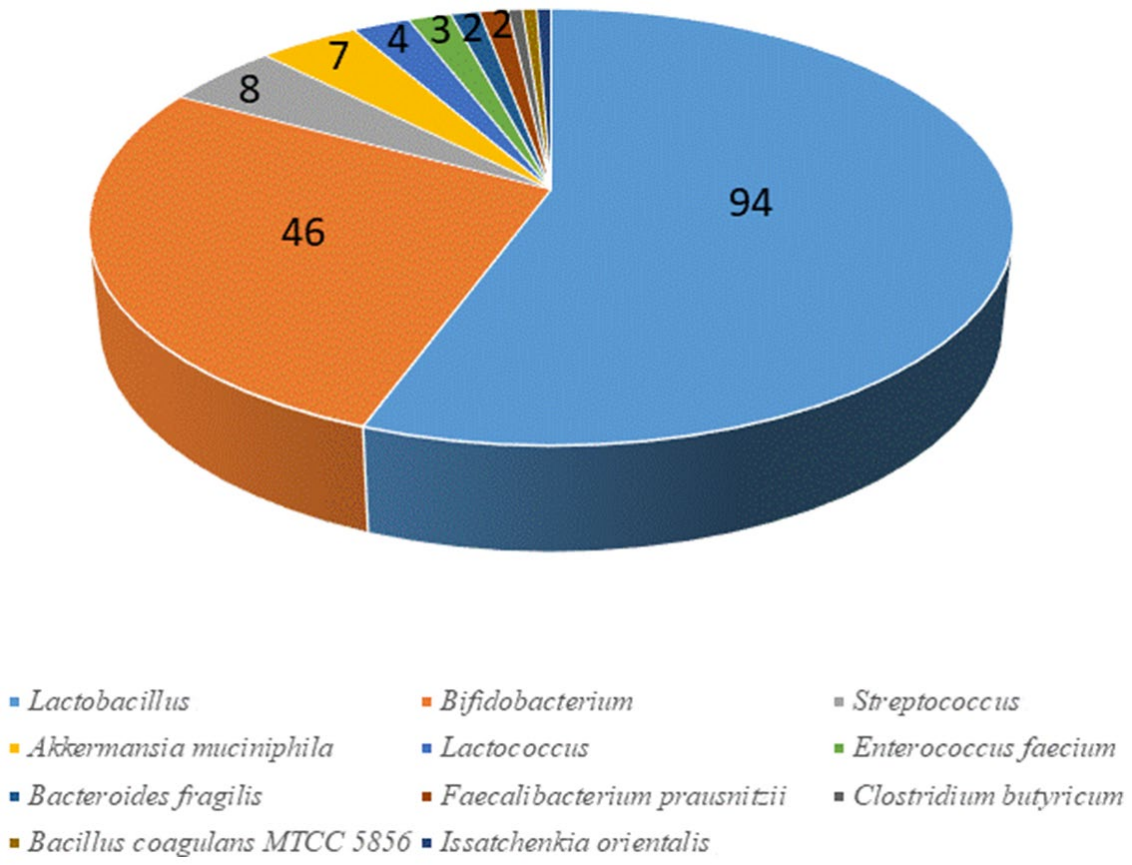
Probiotic genera mostly used in clinical trials.

This review shows that there are significant effects of probiotics on a wide variety of conditions; moreover, a focus at genus/species level on research papers with a robust design beyond and with proven effects (*ca.* 160) suggests the efficacy of lactobacilli (*L. plantarum*, *L. casei*, *L. acidophilus*, *L. reuteri*, among others) and bifidobacteria (*B. longum*, *B. infantis*, *B. animalis*, *B. bifidum* or *B. breve*), with promising evidence for a new generation of probiotics (mainly *A. muciniphila*, *B. fragilis*, and *F. prausnitzii*; Figure 4). Apart from species, the identification of the dose required to gain a measurable output is controversial. Many probiotic supplements contain 1 to 10 billion CFU per dose, up to 50 billion CFU or more; however, higher CFU counts do not necessarily improve health effects. In fact, depending on the disorder, it may happen that even a lower dose can be effective or even better than a higher dose (130). Supplementary Table S1 shows the doses, when available, for the different trials; generally, the concentrations for the most important commercial preparations of *Lactobacillus* spp. and *Lactobacillus* related genera are from 10^9^ to 10^10^ CFU, while for *Bifidobacterium* spp. at 10^8^–10^10^ CFU, for *Pediococcus acidilactici* 10^9^ CFU, for *Streptococcus thermophilus* 10^8^ CFU, for yeast strains such as *Saccharomyces boulardii* 10^9^ CFU, *Bacillus subtilis* 10^9^ CFU and *A. muciniphila* 10^8^ CFU (131). It is worth mentioning that the dose is also a function of storage conditions, as some preparations should be stored at room temperature, while others require refrigeration; therefore, a thermal abuse could heavily affect probiotic survival. The International Scientific Association for Probiotics and Prebiotics advises manufacturers to list expected probiotic concentration on the “expiration” or “use by” date on the product label when stored at proper conditions and suggests consumers to avoid preparations listing the dose of probiotic at the time of production (132).

Strictly linked to the dose, the second critical point is the duration of supplementation, but for this aspect there is not a consensus in the literature; generally, it is believed that probiotics should be assumed for several weeks (at least from 2 to 4 weeks) to gain achievable outputs (133). However, the supplementation could be either short-term or long-term, with short-term interventions suggested only for acute gastro-intestinal conditions (5–7 days for acute diarrhea in infants and children, from 1 to 4 weeks for antibiotic-associated diarrhea, a few weeks for constipation) (133, 134), while other conditions require long-term supplementation, up to 2–3 months for IBD, 3–6 months for Chron disease, atopic dermatitis, or psychiatric diseases (133–136).

Another critical point is the delivery. Probiotics are marketed in different forms such as capsules, tablets, films, or hydrogels, and for oral delivery the microencapsulation in hydroxypropyl methylcellulose phthalate (HPMCP), hydroxypropylmethyl cellulose acetated succinate, and cellulose acetate phthalate (CAP) is used to minimize the exposure of probiotics to gastric acids, reducing their viability loss in the stomach (137). It is a matter of debate if oral delivery mediated by foods could result in a higher impact of probiotics (138), while other ways of delivery, less used at least for the studies reported in this review, are nasal, transdermal, rectal, and vaginal (137).

Also, production could affect viability and thus health effects of probiotics; fermentation is the most common method of producing commercial probiotics: in a large fermentation vessel, single-strain probiotics are inoculated into a liquid broth that is stirred to prevent bacterial settlement and with pH kept under control. When the production concerns anaerobic species, gasses such as nitrogen, hydrogen, and carbon dioxide, are controlled. Microbial growth is controlled by cell density measurements and light/fluorescence microscopes are used to check for unwanted contaminations. Once batch fermentation is complete, a filtered and concentrated cells suspension is either spray-dried or freeze-dried but previously, cryoprotectants or lyoprotectants are added to prevent loss of microbial viability (131).

To increase the production rate the batch fermentation is integrated with crossflow membrane ultra/microfiltration; when the desired cell density is reached, toxic metabolites and/or acids are removed through a membrane. Fresh medium is continuously pumped into the fermenter by varying flow rates to ensure a constant total volume. The cell suspension can then be extracted in batches or continuously (131).

Another effective method to enhance the production of probiotics is the immobilization in natural biopolymers such as protein-based biopolymers, polysaccharides, lipids, and synthetic polymers or coating for the protection of probiotics against moistures or gasses (oxygen/carbon dioxide) (131). Cells are immobilized in polysaccharide hydrogels, then placed in a fermenter, where the medium is regularly supplemented, and the cells periodically removed to ensure proper dilution. This strategy is used to improve overall growth rate and cell viability. The benefits of this approach are the continuous and controlled delivery of probiotics to the gut, a higher viability, and lower costs, while the some limits are the restricted biocompatibility of some immobilization agents, and the complexity of production processes (131).

## 11. Conclusions and perspectives

The use of probiotics could be a promising strategy to counteract side or secondary effects in several pathological conditions; the evidence and data hereby reported suggest a benefit in CRC both as a preventive measure to avoid carcinogenesis or during medical treatments to favor recovery, or in improving cognitive functions, in ameliorating the symptoms of some intestinal diseases (e.g., IBD), or to counteract obesity, diabetes and other metabolic syndromes. The effect is generally mediated through the modulation of gut microbiota, as well as on the production of significant amounts of SCFA, which exert in turn several physiological functions, and the final output could be symptoms amelioration or disease remission, although the use of different clinical outcomes is a challenge, as it makes difficult a comparison of different trials and research papers.

At species level, most data are available on Lactobacillaceae and on *Bifidobacterium* spp., even if evidence is available for *A. muciniphila*, *B. fragilis*, and *F. prausnitzii*. However, there are some issues that should be addressed, related to the duration of the supplementation (short-term or long-term), dose, as each study suggests a different dose (ranging from 10^8^ to 10^10^ CFU). Concerning the way of supplementation, oral delivery is preferred, but there is still a debate on the usefulness of a supplementation through food.

Moreover, most papers focus on the medical point of view, while there is a dark side not addressed, that is the technological story connected to probiotic productions, the way of supplementation (with food or as supplements), the shelf life, and the dose at the time of consumptions, among others. Further efforts are required to address both medical issues and technological/microbiological challenges for an effective use of probiotics as concurrent strategies for many pathological conditions; there are promising evidence and data, but we are still at a preliminary level, as an effective and efficient use of probiotics should be based on the clear definition of a “before” (dose, storage, way of supplementation, duration etc.) and an “after” (outputs clearly evidenced and defined).

## Author contributions

MC, DC, and AB: conceptualization. MS, BS, and AB: methodology. AR, DC, and AB: investigation and data. AB and DC: writing–original draft preparation. DC, AB, BS, AR, MS, and MC: writing–review and editing. MC: supervision. MS and MC: project administration and funding acquisition. All authors have read and agreed to the published version of the manuscript.

## Conflict of interest

The authors declare that the research was conducted in the absence of any commercial or financial relationships that could be construed as a potential conflict of interest.

## Publisher’s note

All claims expressed in this article are solely those of the authors and do not necessarily represent those of their affiliated organizations, or those of the publisher, the editors and the reviewers. Any product that may be evaluated in this article, or claim that may be made by its manufacturer, is not guaranteed or endorsed by the publisher.
