# Supplementary material for: A narrative review on the use of probiotics in several diseases. Evidence and perspectives
Source: Front Nutr. 2023 Jul 10;10:1209238. doi: 10.3389/fnut.2023.1209238 (PMC10368401; doi:10.3389/fnut.2023.1209238)
Supplement: Supplementary file 1 [file Table_1.pdf]

Supplementary Material

The use of probiotics in disease and in several pathological conditions: proven effects, genera mostly used and perspectives

Daniela Campaniello*, Antonio Bevilacqua*, Barbara Speranza, Angela Racioppo, Milena Sinigaglia, Maria Rosaria Corbo**

**** Correspondence:** Maria Rosaria Corbo: mariarosaria.corbo@unifg.it

Table S1: Effects of probiotics

COLORECTAL DISEASE			
**PROBIOTIC STRAINS**	**EFFECTS**	**TARGET**	**REFERENCES**
Bacteroides fragilis	Reduction of inflammation<br>Anticancer effects	Human	(41)
Lactobacillus acidophilus MTCC 5401	Attenuation of intestinal inflammation	Human	(42)
Faecalibacterium prausnitzii	Anti-inflammatory effect	Human	(43)
Lactococcus lactis subsp. cremoris C60	Prevention of CRC development<br>Restoration of T cell populations and regulation of IFN-γ<br>Production of TCD4+ cells	Human	(44)
Lacticaseibacillus casei ATCC334	Inhibition of CRC cell growth<br>Induction of apoptosis by overregulation of the DDIT3 gene	Human	(45)
Butyricicoccus pullicaecorum (10^8 CFU/capsule)	Prevention of necrotic enteritis<br>Reduction of pathogens in the ileum<br>Antitumor effect due to the production of butyrate able to inhibit the growth of CRC cells through overregulation of SLC5A8 and GPR43 genes	Human	(41)
Bifidobacterium longum, Bifidobacterium bifidum, L. acidophilus and *Lactiplantibacillus plantarum* (probiotic mixture $3x10^{13}$ CFU/g)	Reduction of the tendency of CRC cells to migrate in different tissues<br>Reduction of the size of the tumor	Animal model	(46)
Ligilactobacillus salivarius ($1x10^{10}$ CFU/kg of body weight)	Suppression of the dimethylhydrazine (DMH) production<br>Inhibition of cell proliferation and induction of apoptosis in DMH-induced tumor models	Animal model	(47)
Bifidobacterium animalis subsp. lactis HY8002 ($1x10^8$ CFU), *L. casei* HY2782	Improvements in postoperative intestinal dysfunction<br>Increased production of SCFAs<br>Decreased microbes number associated with the development of CRC	Human	(48)

($5x10^7$ CFU) and *L. plantarum* HY7712 ($5x10^7$ CFU) (probiotic mixture)			
L. plantarum CJLP243 (1×10^{10} for 3 weeks)	Improvement on intestinal function and quality of life in patient subjected to rectal resection and admitted to undergo the reversal of the ileostomy<br>Any improvement of symptoms such as diarrhea, fecal incontinence and other complications	Human	(49)
**NEURO-PSYCHIATRIC DISEASES**			
AUTISM (ASD)			
**PROBIOTIC STRAINS**	**EFFECTS**	**TARGET**	**REFERENCES**
Bifidobacterium spp.	Anti-inflammatory effect	Human	(55)
VSL #3 [*L. delbrueckii* subsp. *bulgaricus* ($8x10^{10}$ CFU), *L. acidophilus* ($8x10^{10}$ CFU), *Bifidobacterium breve* ($9x10^{10}$ CFU), *B. longum* ($9x10^{10}$ CFU), *Bifidobacterium infantis* ($9x10^{10}$ CFU), *Lacticaseibacillus paracasei* ($8x10^{10}$ CFU), *L. plantarum* ($8x10^{10}$ CFU), *Streptococcus thermophilus* ($20x10^{10}$ CFU)]	Significant reduction of the main symptoms of autism and severity of gastrointestinal symptoms in boys with ASD and with severe impairment of cognitive function	Human	(56)
L. acidophilus, Lacticaseibacillus rhamnosus, B. longum	Improvement of gastrointestinal disorders<br>Reduction of severity of ASD and ATEC score<br>Alteration of the fecal microbiota of children with ASD with increase of bifidobacteria and lactobacilli	Human	(57)
B. infantis ($2x10^{13}$ CFU for day)	Reduction of IL-13, gastrointestinal symptoms, onset of particular aberrant behaviors in children aged 2 to 11 years with ASD	Human	(58)
Limosilactobacillus reuteri (10^8 CFU(mL), *B. longum* (10^8 CFU/mL)/GOS (5 g)	Increased gastrointestinal resistance, positive modulation of the intestinal microbiota and metabolic activity of children with ASD	Human	(59)

L. plantarum PS128 (3x10^{10} CFU/capsule)	Improvement of ODD (oppositional defiant disorder) and total SNAP-IV scores for children aged 7-12	Human	(60)
L. reuteri (10^{10} CFU/g for day for 3 months)	Improvement of oxytocin-dependent behavior in ASD patients	Human	(61)
L. rhamnosus GG (ATCC 53103) (1x10^{10} CFU daily for 4 week)	Reduction of neuropsychiatric disorders in childhood	Human	(62)
Each packet contained 450 billions of eight probiotics: *S. thermophilus*, *B. breve*, *B. longum*, *B. infantis*, *L. acidophilus*, *L. plantarum*, *L. paracasei*, *L. delbrueckii* subsp. *bulgaricus*	Decrease in total ADOS-CSS from 6.84 to 6.19 in the probiotic group compared to the placebo group	Human	(139)
Lactobacillus spp., *Bifidobacterium* spp., *Streptococcus* spp.	Reduction in the amount of *Desulfovibrio* spp.; normalization of the *Bacteroidetes/Firmicutes* ratio in children's feces	Human	(140)
B. bifidum (1.9×10^8 CFU/g), *B. infantis* (1.9×10^8 CFU/g), *Lactobacillus helveticus* (6.4×10^9 CFU/g)/FOS, Maltodextrin	Decreased incidence of ASD and inhibition of IL-6 and IL-17 production	Animal model	(141)
Bifidobacterium spp., *Lactobacillus* spp. (probiotic mixture 1 billion CFU/sachet or 1 × 10^9 CFU/day)	Improvement of glutamate excitotoxicity with GABA restoration and decrease of excitatory neurotransmitter	Animal model	(142)
ANXIETY AND DEPRESSION			
Akkermansia muciniphila	Antidepressant effect<br>Improvement of intestinal microbiota<br>Increased of BDNF<br>Inhibition of neuroinflammatory response	Animal model	(63)
Mixture of: *B. bifidum* W23, *Bifidobacterium lactis* W52, *L. acidophilus* W37, *Levilactobacillus brevis* W63, *L. casei* W56, *L. salivarius*	Reduction of depressive behavior in FST due to the interaction with immune system, hippocampal HPA axis regulation and microbial tryptophan metabolism	Animal model	(64)

W24, *Lactococcus lactis* W19, *Lc. lactis* W58 (2.5×10^9 CFU/g)			
L. rhamnosus (dosage ranged from $1x10^8$ to $1x10^{10}$ CFU)	Reduction of anxious behaviour in anxiety animals	Animal model	(65)
Lactobacillus spp.	Improvement of behavioural abnormalities	Human	(66)
B. bifidum (2×10^9 CFU/g), *L. acidophilus* (2×10^9 CFU/g), *L. casei* (2×10^9 CFU/g)	Reduction of depression symptoms, of plasma insulin and glutathione	Human	(67)
Lactobacillus spp., *Bifidobacterium* spp.	Antidepressant effects	Human	(68)
L. helveticus, B. longum (ten billion CFU per 5 g sachet.	Reduction of BDI scores (17.39- 9.1) compared to the prebiotic group (19.72-14.14) and the placebo group (18.18-15.55)	Human	(69)
B. bifidum BGN4, *B. longum* BORI (a total of 1×10^9 CFU)	Reduction of intestinal bacteria responsible for inflammation<br>Improved mental flexibility test<br>Increased serum BDNF level, stress relief in the elderly	Human	(70)
L. plantarum P8 ($2x10^{10}$ CFU/sachet/day)	Reduction of anxiety and stress symptoms, plasma cortisol levels<br>Improvement of serotonin,<br>Increased of IL-10	Human	(71)
DEMENTIA AND COGNITIVE DETERIORATION			
Enterococcus faecium (4×10^8 CFU)/Inulin	Improvement in spatial learning and memory, inflammatory state, and levels of BDFN and butyrate	Human	(72)
B. breve A1 (2 capsules daily containing $>2.0\times10^{10}$ CFU)	Improvement of language, attention, guidance in the elderly	Human	(73)
L. casei LC122, *B. longum* BL986 (probiotic mixture of 2×10^9 CFU $mouse^{-1} d^{-1}$)	Improvement of the accumulation of liver lipids, strength and muscle function Attenuation of oxidative stress and inflammation in peripheral tissues Improvement of the intestinal barrier.<br>Better learning and memory skills	Animal model	(74)
Clostridium butyricum ranged from $1x10^6$-$1x10^8$ CFU	Improvement of memory and spatial learning<br>Attenuation of morphological changes of hippocampus and cellular apoptosis Increased in BDNF levels	Animal model	(75)

PARKINSON'S DISEASE			
L. acidophilus, *B. bifidum*, *Lim. reuteri*, *Limosilactobacillus fermentum* *B. bifidum* (2×10^9 CFU/day each)	Positive impact on MDS-UPDRS scores	Human	(76)
L. salivarius, L. acidophilus	Reduction of pro-inflammatory cytokine levels (IL-6, TNF-α, IL-17A) and ROS<br>Increased of anti-inflammatory cytokines (IL-4 and IL-10) in PBMC	Human	(77)
Lactobacillus spp., *Bifidobacterium* spp.	Improvement of intestinal integrity<br>Reduction of anxiety, depression and stress<br>Regulation of GABA receptor expression in SNS	Animal model	(78)
ANOREXIA NERVOSA (AN)			
L. plantarum P8 (10 log CFU daily)	Alleviation of AN related anxiety and stress	Human	(81)
B. fragilis	Correction of gastrointestinal function and restoration of serotonin production	Human	(82)
Lactobacillus spp. (range between $1x10^6$-$2x10^{12}$CFU)	Promotion of weight gain	Animal model	(83)
Lactobacillus spp. (for *L. reuteri* $2x10^{12}$ CFU/mouse/day	Improvement of behavioral abnormalities in stressed mice thanks to the inhibition of the enzyme IDO1 responsible for the conversion of TRP to KYN, achieving a decrease in the circulating level of KYN and modulating the gut-brain axis	Animal model	(84)
A. muciniphila	Restoration of the intestinal barrier, the expression of proteins at a narrow junction, increasing the thickness of mucus. $2x10^8$ bacterial cells per day partly protecting against diet-induced obesity in mice	Animal model	(85)
ALZHEIMER DISEASE (AD)			
L. acidophilus, B. bifidum, B. longum 500 mg probiotics (15×10^9 CFU for 6 weeks).	Improved spatial and memory learning, long-term enhancement and lipid profiles	Animal model	(86)
L. acidophilus, B. bifidum, B. longum (2×10^9 CFU/day each)	Improved cognitive function in AD patients with increased MMSE scores	Human	(87)

SCHIZOPHRENIA			
Lactobacillus rhamnosus GG *Bifidobacterium animalis* subsp. *lactis* Bb12	Improvement of the typical symptoms of schizophrenia	Human	(88)
MULTIPLE SCLEROSIS (MS)			
Mixture of: *L. paracasei, L. plantarum, L. acidophilus, Lactobacillus delbrueckii, B. longum, B. infantis, B. breve, S. thermophilus* (900 billion CFU per sachet)	Improvement of MS symptoms through modulation of the intestinal microbiota and anti-inflammatory immune response in patients with MS	Human	(89)
**INTESTINAL DISEASES**			
INFLAMATORY BOWEL DISEASE (IBD)			
**PROBIOTIC STRAINS**	**EFFECTS**	**TARGET**	**REFERENCES**
F. prausnitzii	Anti-inflammatory effect	Human	(43)
A. muciniphila	Release of vesicles with anti-inflammatory activity	Animal model	(92)
A. muciniphila	Immune and metabolic regulation with increased intestinal barrier	Human	(93)
VSL #3 *(L. casei, L. plantarum, L. acidophilus, L. delbrueckii* subsp. *bulgaricus, B. longum* subsp. *longum, B. breve, B. longum* subsp. *infantis, S. salivarius* subsp. *thermophilus)* (probiotic mixture containing $9x10^{14}$ viable lyophilized bacteria)	Maintaining state of remission	Human	(94)
L. acidophilus	Restored the balance of inflammatory cytokines	Animal model	(95)

IRRITABLE BOWEL DISEASE (IBS)			
Probiotico F1 = *L. acidophilus* (5 × 10^9 CFU), *L. reuteri* (5 × 10^9 CFU) Probiotico F2 = *L. plantarum* (5 × 10^9 CFU), *L. rhamnosus* (5 × 10^9 CFU), *B. animalis* subsp. *lactis* (5 × 10^9 CFU)	In both groups there was an improvement in symptoms such as bloating, abdominal pain, constipation, abdominal cramps and flatulence	Human	(97)
Bacillus coagulans MTCC5856 (tablet containing 2 × 10^9 CFU/day for 90 days)	Improvement of IBS symptoms<br>Reduction of: bloating, vomiting, diarrhea, abdominal pain, stool frequency	Human	(98)
L. plantarum DSM 9843 (5 × 10^7 CFU/mL per day for 4 weeks)	Decrease in pain and flatulence	Human	(99)
L. acidophilus NCFM (10^9 and 10^{10} CFU)	Improvement of IBS symptoms, average reduction of IBS-SSS sum score	Human	(100)
L. rhamnosus, L. plantarum, L. acidophilus, E. faecium (50 mL dose containing 1x10^{13} live bacteria)	Improvement of IBS-SSS scores (from -63.3 87.9 and 28.3 81.2), of IBS symptoms in the probiotic group compared to placebo	Human	(101)
**OBESITY**			
**PROBIOTIC STRAINS**	**EFFECTS**	**TARGET**	**REFERENCES**
Lactobacilllus gasseri SBT2055 (10^8 CFU/g).	Reduction in visceral and subcutaneous fat, body weight and BMI	Human	(103)
L. fermentum (1.08x10^9 CFU microencapsulated), *Lactobacillus amylovorus* (1.39x10^9 CFU microencapsulated)	Reduction in total body fat mass	Human	(104)
S. thermophilus, *L. delbrueckii* subsp. *bulgaricus*, *L. acidophilus* LA-5, *Bf. animalis* BB12	Reduction of total cholesterol, LDL and HDL cholesterol, serum triglyceride concentration	Human	(106)

A. muciniphila	Improvement of inflammation, insulin resistance and blood sugar through the role of epithelial barrier	Human	(108)
A. muciniphila	Reduction of: fat mass, body weight, liver steatosis, inflammation Restoration of the function of the intestinal barrier, glucose resistance	Animal model	(108)
**DIABETES**			
**PROBIOTIC STRAINS**	**EFFECTS**	**TARGET**	**REFERENCES**
L. reuteri DSM 17938 (10^8 CFU/d or 10^{10} CFU/d for 12 weeks).	Increased ISI and DCA levels, related to improved insulin sensitivity<br>Increased microbial diversity	Human	(114)
L. paracasei HII01 (50×10^9 CFU/day for 12 weeks)	Reduction of FBG level, inflammatory markers such as TNF-α and IL-6, and hsCRP in 50 diabetic patients.	Human	(115)
L. rhamnosus LRa05 (10^9 CFU/day)	53.5% FBG and insulin resistance reduction<br>Relief of: liver oxidative stress and metabolic inflammation related to LPS levels in mice with T2DM<br>Increase in bacteria producing SCFA<br>Decrease in proinflammatory bacteria	Animal model	(116)
Camel milk probiotic compound (CPCM)= *Lactobacillus kefiranofaciens*, *L. plantarum*, *L. helveticus*, *Lc. lactis* *Issatchenkia orientalis* (Bacteria at $1x10^8$-$1x10^{10}$ CFU/mL; yeast at:$1x10^6$-$1x10^8$ CFU/mL)	Reduction of FBG, oral glucose tolerance<br>Regulation of intestinal flora disorders with improvement of liver and kidney damage in mice db/db	Animal model	(117)
L. acidophilus ($2x10^9$ CFU)*, L. casei* ($7x10^9$ CFU)*, L. bulgaricus* ($2x10^8$ CFU)*, L. rhamnosus* ($1.5x10^9$ CFU)*, Bf. breve* ($3x10^{10}$ CFU)*, B. longum* ($7x 10^9$ CFU)*, S. thermophiles* ($1.5x10^9$ CFU) / FOS (100 mg)	Reduction of FPG, increase of HDL-C	Human	(118)

METABOLIC SYNDROME			
**PROBIOTIC STRAINS**	**EFFECTS**	**TARGET**	**REFERENCES**
A. muciniphila	Degradation of mucin by stimulating the production of new mucus layer	Human	(108)
A. muciniphila	Immune and metabolic regulation with increased intestinal barrier<br>Production of SCFA	Human	(93)
L. plantarum	Reduction of LDL-C, blood sugar and homocysteine and IL-6 in postmenopausal women	Human	(120)
L. acidophilus, B. bifidum, B. lactis, B. longum (1.5x10^9 for each)/Inulin	Reduction of HDL-C, hyperglycemia and hypertension	Human	(121)
**MUSKULOSCHELETRIC DISEASES**			
**PROBIOTIC STRAINS**	**EFFECTS**	**TARGET**	**REFERENCES**
L. casei	Reduction of pain and destruction of cartilage	Animal model	(125)
L. casei (10^8 and 2x10^9 CFU) *L. acidophilus* (2x10^9 CFU)	Antimicrobial, antioxidant, anti-inflammatory action<br>Reduction of joint swelling and pro-inflammatory cytokines (TNF-α, IL-17A, IL-17F, and IFNγ)<br>Prevention of gastrointestinal dysbiosis and physiological stress	Human	(126)
L. helveticus MTCC 5463 (10^8 CFU/mL), *S. thermophilus* MTCC 5460	Calcium increase	Human	(127)
L. casei Shirota	Recovery of joint strength	Human	(128)
**IMMUNE SYSTEM DISORDERS**			
**PROBIOTIC STRAINS**	**EFFECTS**	**TARGET**	**REFERENCES**
E. faecium	Antitumor action<br>Bactericidal effect against *E. coli*	Human	(95)

	Production of anti-inflammatory cytokines (IL-10, IL-17, and IL-22)		
Li. reuteri 100-23	Development and recruitment of regulatory T cells in the gastrointestinal epithelium	Animal model	(89)
L. rhamnosus HDB1258	Reduction of LPS-induced inflammation in mice through a suppression of LPS-induced TNF-α expression<br>Modification of intestinal microbiota composition with increase of Cyanobacteria and *Bacteroidetes* and reduction of *Deferribacteres*	Animal model	(129)

ADOS-CSS (Autism Diagnostic Observation Schedule – Calibrated Severity Score) = standard assessment tool in autism research;
ATEC = Autism Treatment Evaluation Checklist;
BDI (Beck Depression Inventory) = depression assessment tool;
BDNF = brain-derived neurotrophic factor;
BMI = Body Mass Index;
CRP = C-reactive protein;
CRP = C-reactive protein;
DCA = secondary bile acid deoxycholic acid;
FBG = fasting blood glucose;
FPG = fasting plasma glucose;
FST (Forced Swim Test) = rodent depression assessment test;
GABA = gamma-aminobutyric acid;
HDL = High Density Lipoprotein.
IBS-SSS (Irritable Bowel Disease-Severity Scoring System) = questionnaire to assess the severity of symptoms of IBS
IL = interleukin;
IL = interleukin;
ISI = insulin sensitivity index;
KYN = Kynurenine;
LDL = ; Low Density Lipoprotein;
LPS = lipopolysaccharide,
MDS-UPDRS (Movement Disorder Society-Unified Parkinson's Disease Rating Scale) = Parkinson's disease rating scale.
Mice db/db = mice homozygous for a defect in the leptin receptor;
MMSE (Mini mental State Examination) = cognitive status assessment tool.
ODD, oppositional defiant disorder
PBMC = peripheral blood mononucleate cells;
ROS = reactive oxygen species;
SCFA = short chain fatty acids;
SNAP-IV = Attention Deficit Hyperactivity Disorder (ADHD) assessment test;
SNS = Sympathetic nervous system;
T2DM = Type 2 Diabetes Mellitus;
TNF = tumor necrosis factor;
TRP = Tryptophan.
